# Prevalence of HIV infection and high-risk behaviors in truck and bus drivers in Kurdistan province

**DOI:** 10.1186/s12879-021-06903-0

**Published:** 2021-11-28

**Authors:** Aisan Karimi, Reza Ghanei Gheshlagh, Abdorrahim Afkhamzadeh, Obeidollah Faraji, Khaled Rahmani

**Affiliations:** 1 Kurdistan University of Medical Sciences, Sanandaj, Iran; 2 Spiritual Health Research Center, Research Institute for Health Development, Kurdistan University of Medical Sciences, Sanandaj, Iran; 3 Social Determinants of Health Research Center, Research Institute for Health Development, Kurdistan University of Medical Sciences, Sanandaj, Iran; 4 Liver and Digestive Research Center, Research Institute for Health Development, Kurdistan University of Medical Sciences, Sanandaj, Iran

**Keywords:** Truck driver, Bus driver, HIV/AIDS, Risky sexual behavior, Drug use

## Abstract

**Background:**

Truck, bus, transit drivers, and men with mobile jobs are at high risk for HIV/AIDS. The purpose of this study was to investigate the prevalence of HIV and risky behaviors among truck and bus drivers in Kurdistan province.

**Methods:**

This cross-sectional study was conducted on 601 bus and truck drivers in Kurdistan province during 2018–2019. Data on high-risk behaviors were collected using a standard questionnaire. ELISA test was used to detect HIV in the study participants. Data were analyzed using Chi-square, Mann–Whitney U tests, and logistic regression modeling in Stata-14 software.

**Results:**

The mean and standard deviation of the age of study participants was 44.04 ± 11.44 years. HIV rapid test was positive in two subjects; in other words, the prevalence of HIV in the study population was 0.33%. Ninety-two (15.3%) individuals reported a history of drug use, with one (1.1%) having a history of injecting drugs. One hundred and thirty-one (21.8%) of them had a history of high-risk sexual behavior outside of marriage.

**Conclusion:**

According to the results of the present study, the prevalence of high-risk behaviors in bus and truck drivers is high. It seems necessary to direct the drivers’ attention to self-care while considering disciplinary intervention programs to prevent the use of drugs, cigarettes, alcohol along with high-risk sexual behaviors to maintain the health of drivers and passengers.

## Background

Transportation is an all-encompassing, complex, and dynamic phenomenon that nourishes and enlivens the human economy. In large countries that do not have a rail transport system or their rail transport system is inadequate, the road transportation system is very important [1]. Truck drivers are the main agents of this transportation system, playing a critical role in the trade and logistics industries by transporting goods to local or remote areas [2].

Truck and bus drivers are at risk for drug abuse due to their long-distance driving, busy work schedule, improper rest, and lack of rest [3]. Long working hours [4], sleep deprivation [5], low physical activity [6–8], workplace violence, and road safety hazards [6] lead to increased stress and even a tendency to use amphetamines and drugs in these individuals. On the other hand, these drivers have fewer social restrictions and are more inclined to relation with commercial sex workers and high-risk sexual networks due to the mobile nature of their job and being away from family for a long time [9, 10]. They may not have adequate access to health services and protective devices, including prompt and effective treatment for sexually transmitted infections (STIs), condoms, and other preventative interventions [9, 11]. Accordingly, truck drivers and those who have sex with them can play a role in the spread of HIV/AIDS, hepatitis, and other sexually transmitted infections [12].

According to the existing scientific literature, although many studies have been conducted on some high-risk groups in Iran, such as addicts, injecting drug users (IDUs), prisoners, and sex workers, so far, less attention has been paid to truck and bus drivers as a vulnerable group in terms of HIV infections, high-risk behaviors, sexually transmitted diseases, and social harms. Accordingly, the present study aimed to investigate the prevalence of HIV/AIDS as well as high-risk behaviors in truck and bus drivers in Kurdistan province.

## Methods

This was a descriptive-analytical cross-sectional study. The study population included truck and bus drivers in Kurdistan province, northwest of Iran. Our inclusion criteria were age over 20 years, residence in Kurdistan province, and at least 1 year of experience as a truck, transit, or suburban bus driver. Studied samples included 601 drivers, randomly selected from the list of drivers registered in the Department of Transportation and Terminals of Kurdistan Province. All 601 invited drivers were participants in our research (response rate = 100%). Data collection of this study was done with the cooperation of AIDS experts and consultants of behavioral diseases counseling centers in 10 cities of Kurdistan province and the Department of Transportation and Terminals of the province. The data collection process took 3 months due to the simultaneous absence of all drivers in the province. After explaining the objectives of the project, written consent was obtained from all drivers participating in the study. To comply with ethical standards, the questionnaires were distributed anonymously to individuals and they were assured that all this information would be confidential. The design and protocol of this study were assessed and approved by the Ethics Committee of the Vice Chancellor for Research and Technology in Kurdistan University of Medical Sciences (Registration Number: IR.MUK.REC.1396 0.210).

A checklist was used to collect demographic information, and a standard questionnaire was used to collect data related to high-risk sexual behaviors and substance use, which has been used in Bio-Behavioral Survey studies in Iran [13]. HIV status was also determined using ELISA. For this purpose, a blood sample was taken from each participant after the end of the questionnaire completion, and at the end of the daily work, the samples were transferred to the laboratory of the Reference Behavioral Diseases Counseling Center of the province following the standard conditions. In the laboratory, HIV infection was assessed using fourth-generation ELISA kits.

Data analysis was performed using Stata software version 14. First, the data were summarized using descriptive indicators (mean, standard deviation, frequency, and percentage). The analysis of relationships between different variables with high-risk behaviors (univariate analysis) was performed using the Mann–Whitney U test (due to the abnormality of the age variable), Chi-square, and Fisher’s exact test. Finally, multiple logistic regression modeling was conducted to investigate the relationship between high-risk sexual behavior and significant variables in the initial analysis (univariate) and calculate the values of odds ratio (OR), 95% confidence interval (CI), and significance of each variable.

## Results

In this study, 601 truck and bus drivers of Kurdistan province, whose mean and standard deviation of age were 44.04 ± 11.44 years, were investigated. All subjects were male. The mean and standard deviation of their driving experience was 14.69 ± 9.05 years. The maximum and minimum years of activity as a driver were 45 years and 1 year, respectively. Sixty (10%) of them had a history of imprisonment, with a mean and standard deviation of 6 and 4.21 months, respectively. The minimum length of imprisonment was 10 days, and the maximum was 15 months. Ninety-two drivers (15.3%) had a history of drug use. The mean age of onset of drug use in these individuals was 25.13 years with a standard deviation of 7.73 years, with the lowest and highest age of onset of drug use being 12 and 55 years, respectively. The average years of drug use in the sample was 13.80 years. Among the drivers under study, 515 (85.7%) were married, 72 (12%) were single, and 14 (2.3%) were living apart or divorced. Finally, 49 (8.2%) of the sample had academic education, 543 (90.2%) had diplomas and undergraduates, and 9 (1.5%) were illiterate.

HIV serology test was positive in two subjects. In other words, the prevalence of HIV in the study participants was 0.33%. The high-risk behaviors studied in the subjects included drug use and sexually risky behaviors, the results of which are summarized in Tables 1 and 2. According to the results, 92 (15.3%) of the drivers had a history of drug use, and opium (81.5%) was the most common drug. Forty-two individuals (45.7%) with a history of drug use had used drugs for the first time in friends’ homes, and 76 (82.6%) of them mentioned inhalation as the first method of drug use. Only one person (1.1%) had a history as an injecting drug user. In terms of a history of sexually risky behaviors, 131 (21.8%) of the studied drivers had a history of sex outside of marriage, of which only 12 (9.2%) always used condoms, 70 (53.4%) sometimes, and 49 (37.4%) had never used a condom (Tables 1 and 2).

**Table 1:** Information related to the history of drug use in truck and bus drivers

Variable	Frequency (%)
Drug use history	
Yes	92 (15.3)
No	509 (84.7)
Type of drug use	
Opium	75 (81.5)
Methamphetamine	9 (9.8)
Heroin	3 (3.3)
Methadone	1 (1.1)
Combination of several types of drugs	2 (2.2)
Other (cannabis, tramadol)	2 (2.2)
Location of drug use for the first time	
Friend’s house	42 (45.7)
Private house	27 (29.3)
During driving	13 (14.1)
Prison	2 (2.2)
Other	8 (8.7)
Way of drug use for the first time	
Inhalation	76 (82.6)
Oral	11 (12.0)
Injection	1 (1.1)
Other	4 (4.3)
Current drug use	
Yes	40 (43.5)
No	52 (56.5)

**Table 2:** Information related to the history of sexual risky behaviors in truck and bus drivers

Variable	Frequency (%)
History of sex outside of marriage	
Yes	131 (21.8)
No	470 (78.2)
Condom use, (n = 131)	
Always	12 (9.2)
Sometimes	70 (53.4)
Never	49 (37.4)
Reason for not using a condom, (n = 119)	
No condom available	56 (47.0)
Not willingness to use	45 (37.8)
Sexual partner opposition	2 (1.7)
Not believing in using a condom	14 (11.8)
High cost of getting a condom	2 (1.7)
Partner reason for sex, (n = 131)	
Getting money	29 (22.1)
Love/pleasure	102 (77.9)
The average number of sex outside the marriage per month, (n = 131)	
Less than 5 times	84 (64.1)
5–10 times	17 (13.0)
More than 5 times	30 (22.9)
Trend of the number of sexual partners over time, (n = 131)	
Increased	22 (16.8)
Decreased	66 (50.4)
Without change	43 (32.8)
Sex with married women, (n = 131)	
Yes	70 (53.4)
No	61 (46.6)
History of group sex, (n = 131)	
Yes	13 (9.9)
No	118 (90.1)

Among the studied drivers, 62 (10.3%) had no information on HIV/AIDS, and 527 (87.7%) had not been screened for HIV/AIDS until the time of this study. Ninety-five (15.8%) of them did not believe in reducing the risk of HIV transmission using condoms. Table 3 shows the relationship between the studied variables with the frequency of high-risk behaviors (sex outside of marriage and drug use).

**Table 3:** Relationship between the studied variables and high-risk behaviors (sexually risky behaviors and drug use)

Variable	Sexual risky behaviors		P value	Drug use		P value
	Yes	No		Yes	No	
Marital status						
Married	99 (75.6)	416 (88.5)	**< 0.001	74 (80.4)	441 (86.6)	**0.1
Single	32 (24.4)	54 (11.5)		18 (19.6)	68 (13.4)	
Education						
Illiterate	0	9 (1.9)	***0.3	1 (1.1)	8 (1.6)	***0.3
Diploma and less	121 (92.4)	422 (89.8)		87 (94.6)	456 (89.6)	
Academic	10 (7.6)	39 (8.2)		4 (4.3)	45 (8.8)	
Prison history						
No	106 (80.9)	435 (92.6)	**< 0.001	67 (72.8)	474 (93.1)	**< 0.001
Yes	25 (19.1)	35 (7.4)		25 (27.2)	35 (6.9)	
Sexual risky behaviors						
No	–	–	–	44 (47.8)	423 (83.7)	**< 0.001
Yes	–	–	–	48 (52.2)	83 (16.3)	
Age, Mean ± Sd	38.85 ± 9.96	43.39 ± 10.34	*< 0.001	44.04 ± 11.44	42.10 ± 10.21	*0.1

*Mann–Whitney U test

**Chi-square test

***Fisher’s exact test

According to the results summarized in Table 3, there was no significant difference between the educational status of individuals and the frequency of sexually risky behavior and substance use. The relationship between the variables of substance use and age and marital status was not statistically significant, although the relationship between substance use and the other two variables, history of imprisonment and history of sexually risky behaviors, was statistically significant. Therefore, the relationship between the history of sexually risky behaviors and important variables such as age, marital status, prison history, and substance use was statistically significant. Data were modeled using logistic regression as summarized in Table 4 to better understand the analysis of the relationship between these independent variables and frequency of sex outside the marriage.

**Table 4:** Results of logistic regression modeling to investigate the relationship between sexual risky behaviors and some studied variables

Variable	Unadjusted		Adjusted	
	OR (95% CI)	P value	OR (95% CI)	P value
Age	1.04 (1.02–1.07)	< 0.001	1.06 (1.03–1.08)	< 0.001
Prison history	2.93 (1.68–5.11)	< 0.001	2.24 (1.18–4.24)	0.01
Single vs. married	0.47 (0.28–0.78)	0.003	0.95 (0.51–1.76)	0.8
Not drug use	5.60 (3.49–8.97)	< 0.001	5.97 (3.57–10.0)	< 0.001

*OR* odds ratio, *CI* confidence interval

As mentioned, the variables that had a significant or nearly significant relationship with sex outside the marriage in the univariate analysis (P < 0.1) were modeled using logistic regression. According to the results of this modeling, age, prison history, and drug use had a significant relationship with sexually risky behaviors, i.e., sex outside the marriage. In those who did not use drugs, the chance of having sex outside of marriage was about 6 times higher than those who had used drugs. Also, the chance of having sex outside of marriage was 2.24 times more among those who had a history of imprisonment than individuals who did not have a history of imprisonment.

## Discussion

This study, which aimed to investigate the frequency of HIV/AIDS as well as high-risk behaviors in truck and bus drivers in Kurdistan province, Iran, showed that the prevalence of AIDS in truck drivers was 0.33%. The prevalence of AIDS among truck drivers was 0.1% [14]. The reason for the low prevalence of HIV/AIDS in this occupational group is related to the fact that most of the patients with HIV/AIDS in Iran are injecting drug users, and therefore, contrary to the pattern of other countries, the route of transmission in more than 60% of identified cases in Iran has been a sharing needle among IDUs so far. Although, prevalence of HIV infection in our study participants is relatively low but prevalence of high-risk behaviors among them were high and need to monitor and systematic surveillance. It should be noted that monitoring of risky behaviors among high-risk groups is a part of second-generation HIV surveillance that can provide better data for decision-making and more emphasized by the World Health Organization [15].

The results of a study in India showed that the prevalence of HIV/AIDS, syphilis, and hepatitis B in truck drivers was 15.9%, 13.3%, and 21.2%, respectively [16]. The results of another study in South Africa showed that the prevalence of HIV/AIDS in truck drivers was 26%, and this study also showed that HIV/AIDS infection was associated with spending 2–4 weeks on the road [9]. The prevalence of HIV/AIDS in studies in Africa has been higher than elsewhere. The global prevalence of HIV/AIDS in adults is 1.2%, but it reaches 9% in Africa [17]; therefore, the high prevalence of HIV/AIDS in drivers of African countries, as a part of the general population of those countries, is not far from expectation.

The results of this study showed that 15.3% of the participants had a history of drug use. In another study in Brazil, 5.6% of drivers had a history of drug use [18]. One of the reasons for the high prevalence of drug use among Iranian drivers is the mistakenly justification that drug use can reduce their fatigue and drowsiness. The results of a study by Soori et al., which was conducted to investigate the epidemiology of drug abuse in drivers of suburban public vehicles in Iran, showed that 14.1% prevalence of drug use, which is consistent with the results of our study in Kurdistan [19]. In addition to personal, family, cultural, economic, and social effects, drug abuse by drivers also increases the chances of road accidents, and leads to the death of pedestrians and passengers. According to our data, drug use rate among truck and bus drivers of Iran is relatively high. Since drug use especially among IDUs is a main route of HIV transmission in Iran, some occupational groups such as truck and bus drivers are among high-risk groups for HIV transmission and needing to ongoing monitor and surveillance in these groups is important part of HIV control in the country. During the recent years, Iranian authorities have taken some positive steps to deal with the growing epidemic of HIV/AIDS among IDUs such as harm reduction programs that include providing substitute therapies and establishment of needle/syringe exchanges [20]. The association between imprisonment and drug use is mentioned in several previous studies. Western et al. showed that chance of illicit drug and medication use increases in the year after prison release [21]. In another study conducted by Boys et al. [22], indicated that prisons are a high-risk environment for heroin and other drug initiation and use.

In the present study, 21.8% of drivers had sex outside of marriage, and sexually risky behaviors were more common in married drivers, experienced drivers, and drivers who did not use drugs. Contrary to the findings of the present study, in studies conducted in India and South Africa, the chances of unsafe sex with sex workers were higher in single than in married drivers [9, 23]. In fact, Iranian drivers drive on the roads almost all days of the month due to inflation and high prices, low income, and worn-out cars, and sexual intercourse with their spouses is rare and may increase the chance of unsafe sex with sex workers. The results of Singh et al.’s [24] study showed that 58.8% of truck drivers had sexually risky behaviors). In the study by Ramjee and Gouws [25], 66% of truck drivers had experienced a sexually transmitted infection in the past 6 months. In a study of truck drivers in Uganda, 67.8% had sex with their regular sexual partners, 8.7% with other men, and 33.1% with sex workers [26]. Imprisonment is also can be a risk factor for STDs [27, 28]. Drivers who have a history of imprisonment are not afraid to commit illegal acts due to their bad experiences and ill-treatment of other prisoners. Addiction and unsafe sex with sex workers both seem to be enjoyable and entertaining for drivers, although the addicted drivers are less inclined to have sex because of their higher dependence on drugs and possibly reduced sexual potency.

In this study, only 9.2% of the study subjects used condoms. The results of a study on Pakistani drivers showed that only 6% and 8% of them had used condoms in the past 6 months of their sexual relations with female sex workers and their wives, respectively [29]. It is noteworthy that the study in Pakistan was conducted 18 years ago, and the low use of condoms in the population of truck and bus drivers in Iran is extremely undesirable. In the study of Singh et al. [24], 83.9% of the study subjects used condoms. The results of another study in South Africa showed that 71% and 13% of drivers used condoms for sex with sex workers and their wives, respectively [25].

The current study is almost one of the few studies that has been conducted in the field of HIV and high-risk behaviors in Iranian truck and bus drivers. However, one of the limitations of our study is that we could not investigate alcohol consumption and a history of homosexuality in drivers due to cultural sensitivities and social stigma. Also, despite the attempts to consider the trust of participants, assuring them about confidentiality of their personal information, some drivers may not have reported the actual information due to issues such as fear of unemployment, fear of punishment, and identification.

## Conclusion

Overall, this study showed that prevalence of HIV infection was not high among truck and bus drivers in Kurdistan province, Iran, whereas unsafe sex and drug use were highly prevalent in this group. It is necessary to consider effective harm reduction programs with the aim of preventing the spread of HIV and reducing other harms associated with drug use and sexually risky behaviors among truck and bus drivers.

## Data Availability

The datasets used and/or analyzed during the current study are available from the corresponding author on reasonable request.

## Abbreviations

CI: Confidence interval
OR: Odds ratio
